# Health Information Systems in the COVID-19 Pandemic: A Short Survey of Experiences and Lessons Learned From the European Region

**DOI:** 10.3389/fpubh.2021.676838

**Published:** 2021-09-28

**Authors:** Elsa Negro-Calduch, Natasha Azzopardi-Muscat, Dorit Nitzan, Richard Pebody, Pernille Jorgensen, David Novillo-Ortiz

**Affiliations:** World Health Organization, Regional Office for Europe, Copenhagen, Denmark

**Keywords:** data, health information system, COVID – 19, lessons learned, health data

## Abstract

**Introduction:** The COVID-19 crisis provides an opportunity to reflect on what worked during the pandemic, what could have been done differently, and what innovations should become part of an enhanced health information system in the future.

**Methods:** An online qualitative survey was designed and administered online in November 2020 to all the 37 Member States that are part of the WHO European Health Information Initiative and the WHO Central Asian Republics Information Network.

**Results:** Nineteen countries responded to the survey (Austria, Belgium, Croatia, Czech Republic, Finland, Greece, Iceland, Ireland, Israel, Italy, Kazakhstan, Latvia, Lithuania, Romania, Russian Federation, Sweden, Turkey, United Kingdom, and Uzbekistan). The COVID-19 pandemic required health information systems (HIS) to rapidly adapt to identify, collect, store, manage, and transmit accurate and timely COVID-19 related data. HIS stakeholders have been put to the test, and valuable experience has been gained. Despite critical gaps such as under-resourced public health services, obsolete health information technologies, and lack of interoperability, most countries believed that their information systems had worked reasonably well in addressing the needs arising during the COVID-19 pandemic.

**Conclusion:** Strong enabling environments and advanced and digitized health information systems are vital to controlling epidemics. Sustainable finance and government support are required for the continued implementation and enhancement of HIS. It is important to promote digital solutions beyond the COVID-19 pandemic. Now is the time to discuss potential solutions to obtain timely, accurate, and reliable health information and steer policy-making while protecting privacy rights and meeting the highest ethical standards.

## Introduction

Health information systems (HIS) are systems that incorporate information generated by both population-based and institution-based data sources to provide information to support decision-making (1). The operational response to the COVID-19 pandemic required the rapid adaptation and leveraging of the capabilities of existing HIS to collect, transmit and analyze key health data in real-time that allowed to understand the epidemiological situation and craft appropriate control measures (2). Due to the unprecedented nature of the pandemic in severity and scale, HIS capabilities in many countries were overwhelmed by the information demands and the challenges encountered. Multiple technological gaps were exposed, especially in low and middle-income countries (3, 4). Initial challenges ranged from new demands on key contributors at each health system level, who were already overburdened by the pandemic, to the urgency in determining how to effectively document seamless, continuous COVID-19 processes in electronic health record-embedded (EHR) databases (5).

The WHO Regional Office for Europe (WHO/Europe) unit on Data, Metrics, and Analytics within the Division of Country Health Policies and Systems (WHO/EURO/CPS/DMA) provides the Member States with guidance, tools, and examples of good practices for HIS based on what has worked in the past (6). The COVID-19 pandemic has provided a valuable opportunity to identify the strengths and weaknesses of existing HIS in the context of a global health emergency. Thus, the (WHO/EURO/CPS/DMA) conducted a short qualitative survey to assess Member States' experiences regarding the performance of their national HIS, intending to offer a snapshot of specific concerns, corrective measures adopted, and lessons learned throughout the COVID-19 pandemic.

## Methods

In November 2020, the (WHO/EURO/CPS/DMA) designed and administered an online qualitative survey to assess lessons learned and experiences implementing health information systems (HIS) in the context of the COVID-19 pandemic.

The objectives were to identify experiences, capture valuable insights, and identify issues to be explored further within individual countries. Specifically, we aimed at assessing (1) which components of the HIS worked well, (2) which components of the HIS did not work well, (3) any practical workarounds or solutions, and (4) lessons learned.

The questionnaire included five open-ended questions, one rating scale question, and one yes/no question (Table 1). Open-ended questions were used to gain deeper insights into specific issues and capture responses that would not have been well represented with quantitative data.

**Table 1:** Survey questions.

**No**.	**Question**	**Type**
Q1	Name of the country	Identification
Q2	Name of the person and organization responding to this survey	Identification
Q3	Did existing HIS elements before COVID19 have to be modified to respond to COVID-19 information needs (i.e., clinical case management, public health, and scientific research, etc.)?	Yes/No
Q4	Please comment briefly about the adjustments/modifications/solutions developed.	Open-ended
Q5	Which components of the Health Information System (HIS) for COVID19 have worked well?	Open-ended
Q6	Which components of the HIS for COVID19 do not work so well or had unintended consequences and why?	Open-ended
Q7	Is the country expected to perform any further adjustments to the HIS?	Open-ended
Q8	Has the Health Information System (HIS) in your country responded well to the needs of the COVID19 pandemic (data capture, coding, data use, data analysis, interoperability, etc.)?	0-to-10 rating scale
Q9	What were the lessons learned during the COVID19 pandemic as regards Health Information Systems in your country?	Open-ended

The questionnaire, available in English and Russian, was administered to all the WHO National Focal Points (NFPs) of the 37 Member States of the WHO European Health Information Initiative (EHII) and the WHO Central Asian Republics Information Network (CARINFONET) via a secure internet-based system. The completion time was approximately 10 min to motivate respondents during this busy time and achieve a high response rate. The responses to each question were entered into a Microsoft Excel spreadsheet, combining the datasets from each language. Qualitative data analysis was performed, extracting common traits from the open-ended questions. Where possible, a summary analysis of the quantitative findings of the survey is offered. Results are presented in an aggregated and anonymized format.

## Results

Completed questionnaires were received from 19 out of 37 Member States contacted (51.3% response rate), namely, Austria, Belgium, Croatia, Czech Republic, Finland, Greece, Iceland, Ireland, Israel, Italy, Kazakhstan, Latvia, Lithuania, Romania, Russian Federation, Sweden, Turkey, United Kingdom, and Uzbekistan.

**Figure 1:**
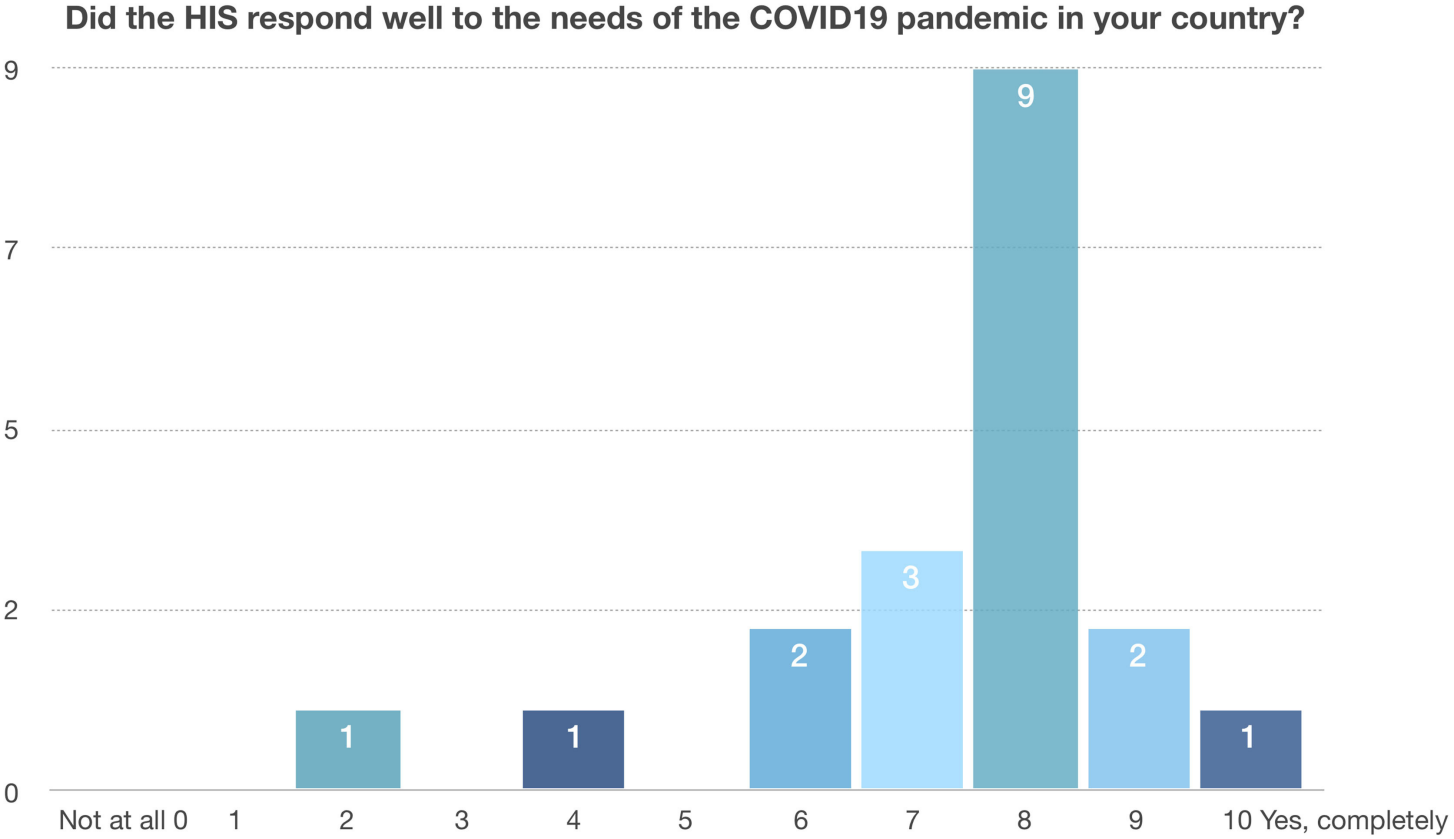
HIS rating scores.

Participants were prompted to rate the HIS COVID-19 response using a 0-to-10 point scale (Question 7). Scores ranged from 2 to 10 with a median score of 8 (interquartile range [25, 75%]: 7, 8). Only two of the 19 countries gave a score below 5 (Figure 1). The median value among all respondents indicates that most respondents felt that the HIS in their countries worked reasonably well and addressed the needs that arose during the COVID-19 pandemic to a satisfactory degree.

Participants were asked to comment on which components of the HIS had worked well (Question 5). The majority (89.4%) indicated that a secure infrastructure for the electronic transmission of health data, already in place, had provided the foundation. In addition, dedicated disease registries, hospital statistics, and mortality registries, maintained over the years, had proven to be valuable data sources for monitoring population health and healthcare provision during the pandemic. Only 11% (*n* = 2) mentioned that the linking of case-based data had been possible. One country indicated that:

“*A National patient portal was already in place and was relatively easy to enhance to provide services to citizens.”*

Others (*n* = 2) commented that reporting to the supranational level had been diligent and in compliance with international standards:

“*The mandatory reporting of clinical cases of some communicable diseases and deaths to a national register, and to the international level (ECDC and WHO) (…) worked well.”*

At the same time, 36.8% (*n* = 7) of participants indicated that HIS had been adapted rapidly:

“*The teams understood the sense of urgency and put everything in place to make things work.”*

“*Within a very short time, a series of surveys and panel studies were established to collect up-to-date data during the crisis.”*

Some countries possessed an existing telemedicine infrastructure before the COVID-19 outbreak, while others developed it during the pandemic to avoid unnecessary patient and staff exposure:

“*e-Prescribing has become more feasible and comprehensive, generating a better capture of patterns and trends and even informing on prescribing patterns and epidemiological data.”*

Two countries set up online workshops to train healthcare workers on COVID-19 clinical information and other instructions, speeding up the implementation of guidelines and protocols.

Regarding adjustments and solutions developed to adapt their HIS to respond to COVID-19 data requirements (Questions 3 and 4), all countries indicated that the existing disease surveillance systems had provided a foundation but needed to be upgraded and reorganized to keep pace with the dynamics of the pandemic. Novel screening processes, hospital-based and ambulatory testing, reporting and analytics tools were all developed or upgraded accordingly to inform public health decision-making:

“*There was an urgent need to develop a system to collect new information - from an emergency preparedness perspective (…). This system was designed specifically as decision support in an emergency and not to collect data for statistics.”*

“*Another main solution developed very quickly was a database containing data on covid-19 patients.”*

“*New dashboards and data pipelines were established to publish updated statistics on cases, deaths, health care and testing.”*

“*The hospital discharge registry was modified to include COVID-19 variables.”*

“*New information systems had to be set up rapidly, e.g., contact tracing information systems and ICU information systems.”*

“*A Public Health Management System (…) was integrated with the entire health system (…) and used at the border gates. Citizens brought to our country from abroad were recorded in this system.”*

“*First rollout of a, albeit temporary, unique patient identifier. The first in the country to be used.”*

Increased reporting frequency (i.e., hospital statistics, prescribed drugs) was cited by 21% (*n* = 4). Twenty-six percent (*n* = 5) mentioned the establishment of new death registration systems to allow for timely calculation of excess mortality:

“*We moved to an electronic and more timely death registration system.”*

Sixteen percent (*n* = 3) of respondents explained that their national version of ICD-10/11 had been quickly updated as soon as COVID-19 coding advice (1) and WHO/ECDC case definitions and recommendations were available (2, 3):

“*We were successful to quickly update the (…) version of ICD-10 when WHO issued coding and terminology recommendations for covid-19 early 2020, and to spread instructions to health care facilities through well-established networks.”*

Eleven percent (*n* = 2) indicated that they were exploring ways to facilitate access and usability of data for research purposes.

The majority of the countries (89%) reported that further adjustments to the HIS were still expected (Question 7). In this regard, two countries specified that additional improvements were anticipated to support the rollout of vaccination programs by setting up national electronic immunization registries.

Most respondents (89.5%) believed that the main issues were the lack of the required data infrastructure for effective information management and accurate reporting on relevant COVID-19 data (Question 6). Dedicated HIS components needed to be upgraded or set up from scratch, often in an uncoordinated manner due to the urgency, imposing a heavy burden on those involved:

“*Covid-19 imposed a heavy burden on both data providers and producers of statistics.”*

“*Increased reporting frequency (i.e., hospital statistics) brought the downside of allowing less data quality control compared to working on a more spaced basis.”*

A transition period was necessary to achieve well-functioning operational processes because of the consequent technical glitches and delays in data reporting. There were instances of suboptimal data capture, poor timeliness, and limited use of information for action by decision-makers:

“*The lack of interoperability and a comprehensive EHR (…) did not allow for sound planning in terms of resources allocation.”*

“*Huge engagement for establishing timeliness, limited use of data at the decision-making level, insufficient interoperability between health care providers and public health authorities”*

Apart from delays related to upgrading HIS components to respond to COVID-19, 31.5% (*n* = 6) of respondents mentioned that a significant factor impacting timeliness, quality, and completeness of data was related to poor interoperability, as well as (in some cases) decentralized HIS operating in different regions or states. These led to problems in coordination, data exchange, and linkage of data:

“*The coordination between agencies and regional/local health authorities could be improved.”*

“*The number of tests, cases in long term care and infected staff were only available on a provincial level.”*

“*The lack of information from primary care settings and municipal health care had a negative impact on our ability to fully assess the interventions during the pandemic.”*

“*It was very difficult to obtain data from the residential and nursing homes, especially from the private ones.”*

“*Existing problems such as the fragmentation of data in several data silos led to problems during the pandemic.”*

“*Largest problems were timeliness and linkage of data.”*

Registration delays on mortality statistics were also reported to have biased the results of excess mortality analyses. For example, one country mentioned that the usual time between a death occurring and being available for excess mortality analysis was three months at the beginning of the pandemic:

“*The national health registries, the causes of death registry, and other individual based registries (…) were not primarily designed to fulfill the more acute needs of emergency surveillance during a pandemic.”*

“*Time lags in mortality data (…) hampered estimates of excess deaths early in the pandemic.”*

Furthermore, one country noted that a large amount of health data was being captured in unstructured clinical notes, making it much more difficult to process and analyze. Thirty-seven percent (*n* = 7) of respondents noted that critical IT infrastructure and labor for effective contact-tracing were insufficient or non-existent before COVID-19. Tools for cluster identification and geo-localization, interpretation, and application of the General Data Protection Regulation (GDPR) were not in place. These were also deemed an important barrier for implementation:

“*The legal aspects and GDPR (interpretation/application) have been a barrier.”*

“*There were some challenges to balance the demand for timely HIS information vs. the need to prevent unauthorized access to confidential information.”*

One NFP reported that resources had been primarily allocated to COVID data collection, negatively impacting effective information management for other diseases:

“*The IT resources allocated to COVID data collection had a negative impact on other data collections.”*

Another respondent mentioned that due to the dramatic increase in the general public and media interest in COVID-19 epidemiology, HIS professionals had to communicate more clearly and widely about data collection specifications, data analysis, and interpretation for different purposes.

Finally, NFPs were asked to elaborate on experiences and lessons learned throughout the COVID-19 pandemic (Question 9). The consensus across the sample was that information needs in an emergency vs. general public health or health system monitoring were very different, and the existing HIS processes and protocols had been developed to serve the latter. Comments from survey respondents are shown in Box 1.

Box 1 **Lessons learned: comments from survey respondents.**

- “*The timeliness aspect is central, and the demand for rapid data capture, analysis and response is quite different in an emergency scenario such as the covid-19-pandemic, compared to the general health system monitoring”*
- “*There is still a lot of work to do to improve data capture, timeliness and interoperability of different information systems”*
- “*The dashboard has been especially successful as a transparency tool”*
- “*Coordinated communication efforts to the political level, the general public and media are essential as the final output from any surveillance system”*
- “*Development of information systems needs good coordination to ensure good interoperability across the health sector”*
- “*Planning and systematic approach in building Health Information Systems were far from desired”*
- “*Advanced HIS is a fundamental component for both expertise advise/evidence, policy development and political action”*
- “*Strong and competent legal teams are needed to quickly assess new situations and to support actions in any area, including information management”*
- “*There is a need for clarifying the application and limits of existing laws governing privacy during the emergency”*
- “*Constant investment and funding will be required for the health information system going into the future”*
- “*Underinvestment in public health administration and in public health research has a negative effect on pro-active interventions”*
- “*Better use of health data for secondary purposes, linkage, sharing and accessing will become the norm due to COVID”*

## Discussion

This brief qualitative research describes how countries in the WHO European Region experienced HIS challenges brought by the pandemic. The limitations of this research relate to the lack of a quantitative approach that would have allowed the measurement of HIS performance by quantifying the distributions of given variables. We preferred a qualitative approach which allowed us to explore the countries' experiences, perceptions, and understanding and determine divergent and common traits from COVID-19 responders at a national level. The survey was designed to be responded in a few minutes to encourage participation, considering COVID-19 priorities. We also hypothesized that providing response options in a more structured questionnaire could have led to acquiescence bias; that is why many of the questions were open-ended. Furthermore, the COVID-19 pandemic is ongoing, and consequently, our assessment captured respondents' perceptions at a single point in time. Although only a bit more than half of the countries (51.3 percent) chose to participate, those which responded represented a wide geographical and economic range.

Information needs during public health emergencies are different from routine health monitoring, and existing HIS were developed to serve the latter (7). The pandemic prompted a greater need for accurate and timely epidemiological data on various topics to understand the impact and plan for an adequate response (8). The capabilities of HIS in every country underwent corrections and enhancements to collect these COVID-19-related data. Typically, HIS upgrades encompass budgeting, planning, design, project oversight, pretesting, communication with end-users, and, finally, implementation (9). However, due to the urgency of the situation, insufficient material and human resources, and lack of proper strategic planning, these stages were improvised or completely skipped, resulting, in some cases, in inadequate data for the COVID-19 information needs and implementation delays. These challenges forced countries to face the limitations of their HIS, raising awareness of the relevance of such systems in public health emergencies. In any case, overall, countries reported satisfaction in how their systems had reacted to the changes in workload, information density, and typology of data.

Social, economic, and cultural differences also shaped how different information strategies coped with the COVID-19 outbreak (10). While some countries had a more developed informatics framework resulting from previous HIS enhancements, others lacked appropriate health information infrastructures capable of meeting the COVID-19 information needs. The pandemic has also exacerbated existing inequalities across HIS globally and highlighted their weaknesses. Although funding was released to support HIS during the emergency, the systems should be prepared for any health crisis in advance (4). Unfortunately, COVID-19 will not be the last global health emergency; thus, it is paramount that both regular funding and government support are secured to continue the implementation and improvement of health information management (11).

The COVID-19 pandemic accelerated the adoption of new health information technologies, and a wide array of digital tools were developed to address health information needs (12–14). For example, the Internet of Things (IoT) provided new data sources. Big data, such as location-based and contact tracing data, were integrated to model epidemiological trends, providing key information to decision-makers (15). However, some of these digital tools brought concerns related to national standards, access, acceptability, usability, adoption, and data protection (2). The General Data Protection Regulation (GDPR) (16) and the ePrivacy Directive (17) provide the safeguards for personal data protection in the European Union. The GDPR states that apps should not identify the individual, and no geolocation or movement data should be used (18). In Norway, “Smittestopp,” the COVID-19 contact-tracing app, was discontinued on 15 June 2020 after receiving a warning from the Norwegian Data Protection Authority (19). Likewise, the UK government was forced to abandon a centralized coronavirus contact-tracing app due to technical (i.e., unsupported by some devices, inaccurate distance measures) and personal privacy concerns (20). In addition, some of the new digital tools that the pandemic has brought have focused on the interests of organizational stakeholders without considering important ethical, social, and cultural values. Despite rapid increases in digital adoption, mobile phone ownership is not equally embraced by all nations. Global mobile users are still under 67 percent of the population (21). Thus, mobile phone location records will not capture these non-mobile phone users (i.e., lower-income, elderly, marginalized groups) (22). These issues need to be reassessed to support information management while meeting the highest ethical standards during health emergencies.

Despite data dashboards being mentioned only by two participants, these have been extensively used to display relevant COVID-19 data (14). However, it is important to note that several facets of a dashboard can be misrepresented without background knowledge of how the data were originally captured, characteristics of the data, and any biases that might affect interpretation (23).

Some survey respondents identified the lack of interoperability as a critical issue, highlighting the importance of the timely exchange of health information across platforms. Integration of multiple data sources remains challenging despite decades of technological advances. Some of the barriers to interoperability include lack of standards, large amounts of unstructured data (8), data breaches, and mistrust (24). There are promising uses for blockchain technology for system integration, specifically in combination with standards for exchanging healthcare information electronically; however, challenges such as immaturity, high cost, data privacy, poor scalability, and low general performance still need to be addressed (24).

Coordination and data sharing have been particularly challenging in countries with a high degree of regional and local decentralization in their health care and social protection and welfare services. Furthermore, coordination and data exchange also need to be improved between organizations within and outside of the health system (i.e., education, internal affairs, etc.).

The COVID-19 pandemic has also stressed the need to tackle infodemics and find efficient ways to communicate and engage with the population to establish trust in public health officials and the information they provide. Coordinated communication efforts to the political ranks, the public, the media, and between agencies and regional and local health authorities are essential, as knowledge translation is the final output from any surveillance system. The HIS-related issues that emerged during the COVID-19 pandemic need to be addressed by responsible information technology research. Developing a holistic view of complex data ecosystems involves the engagement of various data entities in the research process to allow integration and interoperability (22). Also, questions about the usefulness, applicability, and ethical aspects of some digital surveillance technologies still need to be addressed.

## Conclusion

Health information systems with their multiple stakeholders have been put to the test, and valuable experience has been gained. Critical gaps have been revealed, such as under-resourced public health services, obsolete health information technologies, and a lack of interoperability to enable seamless data exchange among disparate organizations within the healthcare sector and administrative divisions. The COVID-19 pandemic has provided an opportunity to recognize and close those gaps to ensure better preparedness against future health emergencies.

Adequate financing into out-of-the-box data management systems is needed. People-centered, cradle-to-grave digitized health records that are seamless across health services and shared with public health and social services are key elements for better policy-making.

The advancements made in artificial intelligence and machine learning can potentially establish linkages between animal, environmental, and human health perspectives, ensuring quality health data and accurate information while respecting privacy rights.

The foundation of quality health data is one of the signs of mature health systems, along with universal health coverage and well-functioning community health and social services. The WHO European Region continues to support countries in developing the health information systems of the future.

## Data Availability Statement

The datasets generated for this study will not be made publicly available due to maintaining confidentiality of identifiable country data.

## Author Contributions

All authors contributed sufficiently and meaningfully to the manuscript's conception, design, drafting, editing, revising, approved the final version for submission, and agreed to be accountable for all aspects of the work.

## Author Disclaimer

The authors alone are responsible for the views expressed in this publication, and those views do not necessarily represent the views, decisions, or policies of the World Health Organization.

## Conflict of Interest

The authors declare that the research was conducted in the absence of any commercial or financial relationships that could be construed as a potential conflict of interest.

## Publisher's Note

All claims expressed in this article are solely those of the authors and do not necessarily represent those of their affiliated organizations, or those of the publisher, the editors and the reviewers. Any product that may be evaluated in this article, or claim that may be made by its manufacturer, is not guaranteed or endorsed by the publisher.

## References

[1] Health Metrics Network. Guidance for the health information systems (HIS) strategic planning process. (2009) Available online at: https://www.measureevaluation.org/his-strengthening-resource-center/resources/GuidancefortheHealthInformationSystemsHISStrategicPlanningProcess.pdf. (accessed May 31, 2021)

[2] Liu C. Health information systems amid COVID-19 outbreak: lessons from China. Health Inf Manag. (2020) 50:99–100. 10.1177/1833358320947557 32806959

[3] Mahmood S Hasan K Colder Carras M Labrique A. Global preparedness against COVID-19: we must leverage the power of digital health. JMIR Public Health Surveill. (2020) 6:e18980. 10.2196/18980 32297868 PMC7164944

[4] Schmidt AE Abboud LA Bogaert P. Making the case for strong health information systems during a pandemic and beyond. Arch Public Health. (2021) 79:13. 10.1186/s13690-021-00531-5 33514433 PMC7844779

[5] Madhavan S Bastarache L Brown JS Butte AJ Dorr DA Embi PJ. Use of electronic health records to support a public health response to the COVID-19 pandemic in the United States: a perspective from 15 academic medical centers. J Am Med Inform Assoc. (2021) 28:393–401. 10.1093/jamia/ocaa287 33260207 PMC7665546

[6] Support tool to assess health information systems and develop and strengthen health information strategies. WHO/Europe. (2017). Available online at: https://www.euro.who.int/en/publications/abstracts/support-tool-to-assess-health-information-systems-and-develop-and-strengthen-health-information-strategies. (accessed May 30, 2021)

[7] Sittig DF Singh H. COVID-19 and the need for a national health information technology infrastructure. JAMA. (2020) 323:2373–4. 10.1001/jama.2020.7239 32421178

[8] He W Zhang ZJ Li W. Information technology solutions, challenges, and suggestions for tackling the COVID-19 pandemic. Int J Inf Manage. (2021) 57:102287. 10.1016/j.ijinfomgt.2020.102287 33318721 PMC7724285

[9] World Health Organization PATH. Planning an Information Systems Project: A Toolkit for Public Health Managers. Seattle: PATH (2013). Available online at: https://path.azureedge.net/media/documents/TS_opt_ict_toolkit.pdf

[10] Haug N Geyrhofer L Londei A Dervic E Desvars-Larrive A Loreto V. Ranking the effectiveness of worldwide COVID-19 government interventions. Nat Hum Behav. (2020) 4:1303–12. 10.1038/s41562-020-01009-0 33199859

[11] Gerke S Stern AD Minssen T. Germany's digital health reforms in the COVID-19 era: lessons and opportunities for other countries. NPJ Digit Med. (2020) 3:94. 10.1038/s41746-020-0306-7 32685700 PMC7351985

[12] World Health Organization. Digital tools for COVID-19 contact tracing. World Health Organization (2020). Available online at: https://www.who.int/publications/i/item/WHO-2019-nCoV-Contact_Tracing-Tools_Annex-2020.1. (accessed May 23, 2021)

[13] Fagherazzi G Goetzinger C Rashid MA Aguayo GA Huiart L. Digital Health Strategies to Fight COVID-19 worldwide: challenges, recommendations, and a call for papers. J Med Internet Res. (2020) 22:e19284. 10.2196/19284 32501804 PMC7298971

[14] Budd J Miller BS Manning EM Lampos V Zhuang M Edelstein M. Digital technologies in the public-health response to COVID-19. Nat Med. (2020) 26:1183–92. 10.1038/s41591-020-1011-4 32770165

[15] Ye Q Zhou J Wu H. Using information technology to manage the COVID-19 pandemic: development of a technical framework based on practical experience in China. JMIR Med Inform. (2020) 8:e19515. 10.2196/19515 32479411 PMC7282474

[16] EU General Data Protection Regulation (GDPR): Regulation (EU) 2016/679 of the European Parliament and of the Council of 27 April 2016. In: EUR - LEX [Internet]. (2016) Available online at: https://eur-lex.europa.eu/eli/reg/2016/679/oj. (accessed Jun 3, 2021)

[17] EU Directive on privacy and electronic communications: Directive (EU) 2002/58/EC. In: EUR-LEX [Internet]. (2009) Available online at: https://eur-lex.europa.eu/legal-content/EN/TXT/?uri=CELEX%3A02002L0058-20091219. (accessed Jun 3, 2021)

[18] Coronavirus: EU interoperability gateway for contact tracing and warning apps. In: European Commission [Internet]. (2020) Available online at: https://ec.europa.eu/commission/presscorner/detail/en/QANDA_20_1905. (accessed May 24, 2021)

[19] Ursin G Skjesol I Tritter J. The COVID-19 pandemic in Norway: the dominance of social implications in framing the policy response. Health Policy Technol. (2020). 10.1016/j.hlpt.2020.08.004 PMC7452841 32874857

[20] Sabbagh D Hern A. UK abandons contact-tracing app for Apple and Google model. The Guardian. (2020). Available online at: http://www.theguardian.com/world/2020/jun/18/uk-poised-to-abandon-coronavirus-app-in-favour-of-apple-and-google-models. (accessed May 24, 2021).

[21] We Are Social Hootsuite. Digital 2021: Global Overview Report. DataReportal – Global Digital Insights (2021) Available online at: https://datareportal.com/reports/digital-2021-global-overview-report. (accessed Aug 9, 2021)

[22] Pan SL Zhang S. From fighting COVID-19 pandemic to tackling sustainable development goals: An opportunity for responsible information systems research. Int J Inf Manage. (2020) 55:102196. 10.1016/j.ijinfomgt.2020.102196 32836647 PMC7338030

[23] Global and European Dashboards Mapping the Spread of COVID-19. In: Data.Europa.Eu [Internet]. (2020) Available online at: https://data.europa.eu/en/impact-studies/covid-19/global-and-european-dashboards-mapping-spread-covid-19. (accessed May 24, 2021)

[24] Chukwu E Garg L. A systematic review of blockchain in healthcare: frameworks, prototypes, and implementations. IEEE Access. (2020) 8:21196–214. 10.1109/ACCESS.2020.2969881

